# 5-Aminolevulinic Acid-Mediated Sonodynamic Therapy Inhibits RIPK1/RIPK3-Dependent Necroptosis in THP-1-Derived Foam Cells

**DOI:** 10.1038/srep21992

**Published:** 2016-02-25

**Authors:** Fang Tian, Jianting Yao, Meng Yan, Xin Sun, Wei Wang, Weiwei Gao, Zhen Tian, Shuyuan Guo, Zengxiang Dong, Bicheng Li, Tielei Gao, Peng Shan, Bing Liu, Haiyang Wang, Jiali Cheng, Qianping Gao, Zhiguo Zhang, Wenwu Cao, Ye Tian

**Affiliations:** 1 Department of Cardiology, the First Affiliated Hospital, Cardiovascular Institute, Harbin Medical University, Harbin, 150001, P. R. China; 2 Department of Pathophysiology and the Key Laboratory of Cardiovascular Pathophysiology, Harbin Medical University, the Key Laboratory of Cardiovascular Research (Harbin Medical University), Ministry of Education, Harbin 150081, P. R. China; 3 Heilongjiang Academy of Medical Sciences, Harbin 150086, P. R. China; 4 Laboratory of Photo- and Sono-theranostic Technologies and Condensed Matter Science and Technology Institute, Harbin Institute of Technology, Harbin 150080, P. R. China; 5 Department of Forensic Medicine, Harbin Medical University, Harbin 150001, P. R. China; 6 Department of Vascular Surgery, the First Affiliated Hospital of Harbin Medical University, Harbin 150001, P. R. China; 7 Materials Research Institute, The Pennsylvania State University, University Park 16802, PA, USA

## Abstract

Necroptosis, or programmed necrosis, contributes to the formation of necrotic cores in atherosclerotic plaque in animal models. However, whether inhibition of necroptosis ameliorates atherosclerosis is largely unknown. In this study, we demonstrated that necroptosis occurred in clinical atherosclerotic samples, suggesting that it may also play an important role in human atherosclerosis. We established an *in vitro* necroptotic model in which necroptosis was induced in THP-1-derived foam cells by serum deprivation. With this model, we demonstrated that 5-aminolevulinic acid-mediated sonodynamic therapy (ALA-SDT) inhibited necroptosis while promoting apoptosis. ALA-SDT activated the caspase-3 and caspase-8 pathways in foam cells, which is responsible for the switch from necroptosis to apoptosis. The inhibition of either caspase-8 or caspase-3 abolished the anti-necroptotic effect of ALA-SDT. In addition, we found that caspase-3 activation peaked 4 hours after ALA-SDT treatment, 2 hours earlier than maximal caspase-8activation. Taken together, our data indicate that ALA-SDT mediates the switch from necroptosis to apoptosis by activating the caspase-3 and caspase-8 pathways and may improve the prognosis of atherosclerosis.

Foam cells, a hallmark of atherosclerosis, accumulate in the intima of arterial wall and form atherosclerotic plaques^1^,^2^. With the progression of atherosclerosis, some of foam cells undergo necrosis and make up necrotic core in the centre of atherosclerotic plaque^3^. Necroptosis, a type of programmed cell death, contributes to necrotic core formation and enhances inflammation, thereby aggravating atherosclerosis in animal models^4^. Hence, targeting necroptosis might be a novel methodology for atherosclerosis treatment.

The concept of necroptosis was introduced in 2005 and demonstrated in cultured cells^1^,^2^. This type of cell death occurs when caspase-8 function is compromised upon overwhelming stress and is defined as caspase-independent programmed cell death^3^,^4^,^5^. In contrast to apoptosis or necrosis, necroptosis is characterized by swollen organelles, disintegrated plasma membrane and intact nuclear membrane^6^,^7^. The finding of necroptosis revises the traditional notion of cell death, in which necrosis is described as an unregulated, passive process. This finding also leads to re-examining the role of cell death in atherosclerosis and other inflammatory diseases^8^,^9^,^10^.

Under the condition of dysfunctional apoptotic signaling, protein kinase RIPK1 (receptor interacting protein kinase 1) and RIPK3 (receptor interacting protein kinase 3) auto- and transphosphorylate each other, and form necrosome. The necrosome then phosphorylates the pro-necroptotic protein MLKL (Mixed Lineage Kinase domain-Like), and phosphorylated MLKL initiates the necroptosis by forming oligomerization and inserting itself into the membranes of organelles and plasma membrane^11^,^12^,^13^,^14^. Genetic deletion of RIPK1, RIPK3 or MLKL, inhibits necroptosis in the multiple mouse models of inflammatory diseases^8^,^13^,^15^. In addition, Necrostatin-1, an inhibitor of RIPK1, is extensively used to prevent necroptosis in preclinical research. Although these methodologies showed efficacy in slowing down the progression of necroptosis-associated diseases in basic research, their clinical benefits and safety require further evaluation. So far, the treatment of necroptosis-related diseases^16^,^17^,^18^,^19^ remains hampered by the lack of an ideal therapy^16^,^17^,^20^.

With strong tissue-penetrating and regional focusing characteristics, SDT selectively kills inflammatory or tumour cells^21^,^22^,^23^. Its preliminary application in cancer patients showed therapeutic benefits^23^,^24^,^25^,^26^. Previous studies in our laboratory have demonstrated that ALA selectively accumulates in macrophages and foam cells in atherosclerotic plaques^27^,^28^,^29^. We also showed that ALA-mediated SDT (ALA-SDT) stabilises atherosclerotic plaques by eliminating foam cells and preventing extracellular matrix degradation, displaying a strong potential for atherosclerosis treatment^27^. However, the underlying mechanism of ALA-SDT remains elusive. In this study, we found that ALA-SDT inhibits necroptosis and enhances apoptosis in THP-1-derived foam cells by activating the caspase-3 and caspase-8 pathways. Switching necroptosis to apoptosis by ALA-SDT may account for its beneficial effects in atherosclerosis treatment.

## Results

### Necroptosis occurs in human atherosclerosis.

**Figure 1:**
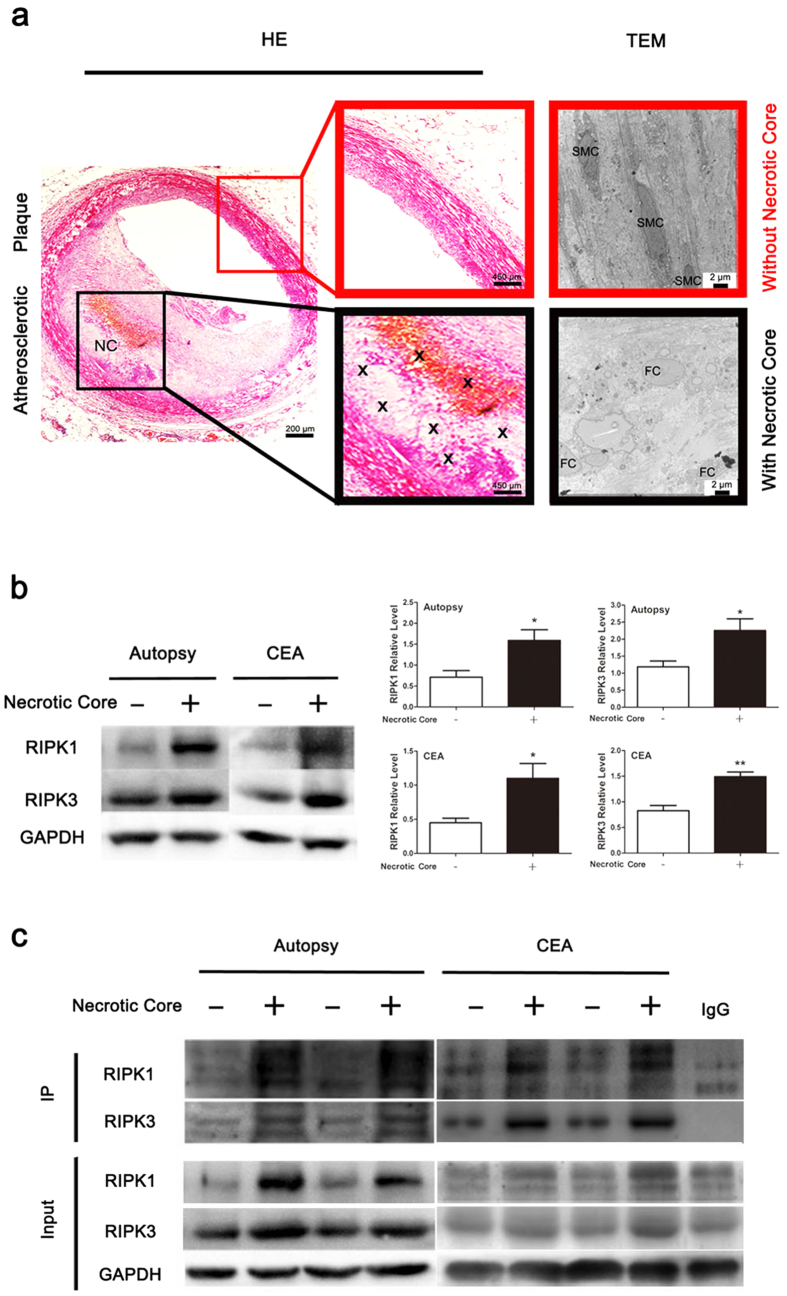
Morphological and biochemical features of necroptosis in human atherosclerosis. (**a**) Representative images showing human atherosclerotic plaques by H&E staining and TEM (scale bars = 200 μm). Magnified insets represent the atherosclerotic plaque without a necrotic core (NC, in red frame), and the atherosclerotic plaque with a necrotic area (in black frame). An “X” indicates a necrotic area in the atherosclerotic plaques (scale bars = 450 μm). The right panel represents TEM images of atherosclerotic plaques with or without a necrotic core (scale bars = 2 μm). TEM image in red frame shows multiple sheets of smooth muscle cells (SMC) in atherosclerotic plaque without a necrotic core. TEM image in black frame indicates cellular composition of atherosclerotic plaque with a necrotic core, and shows the typical necroptotic morphology of foam cells (FC), including a disrupted membrane, translucent cytoplasm, swollen organelles and intact nucleus. (**b**) Western blotting analysis of RIPK1 and RIPK3 levels in atherosclerotic plaque tissues with or without a necrotic core. Protein expression was quantified with reference to GAPDH. (n ≥ 6. *P < 0.05 and **P < 0.01 compared to the group without a necrotic core). (**c**) Immunoprecipitation assay indicates the increase of RIPK1-RIPK3 complex in atherosclerotic plaques with necrotic core. The data represent means ± S.E.M.

Foam cell necroptosis occurs in atherosclerotic lesions and contributes to advanced atherosclerosis in animal models^8^. However, to date, evidence showing that necroptosis exists in human atherosclerotic plaques is missing. To investigate whether this type of cell death takes place in human atherosclerotic plaques, we collected human atherosclerotic tissues from 12 autopsies and 6 patients undergoing carotid endarterectomy (CEA) (Supplementary Figure e). Compared with normal arterial wall in the same section, arterial wall with atherosclerotic lesions was grossly thickened, exhibiting discernible lipid-containing plaque. Based the observation with haematoxylin-eosin (HE) staining, atherosclerotic plaques were separated into two types, with or without a necrotic core (Fig. 1a). For the plaques with necrotic cores, necrosis primarily occurred at the centre of atheroma (Fig. 1a). To determine the cell composition of atherosclerotic plaque, we relied on transmission electron microscopy (TEM). In the plaques without necrotic cores, the main cellular components were smooth muscle cells, which migrate and proliferate from media to the intima responding to arterial wall damage. In contrast, plaques with necrotic cores were full of lipid-rich foam cells, and some of the foam cells around the necrotic core displayed typical necroptotic morphology^6^, including disrupted membrane, translucent cytoplasm, swollen organelles and intact nucleus (Fig. 1a). The protein levels of RIPK1/3 are positively correlated with the extent of necroptosis^30^. Therefore, we examined the expression levels of RIPK1 and RIPK3 in atherosclerotic lesions. Western blotting analysis showed that the levels of these two proteins were significantly increased in atherosclerotic tissues with necrotic cores compared with plaques without necrotic cores (Fig. 1b). Consistent with the western blotting results, the levels of the RIPK1-RIPK3 complex were dramatically elevated (Fig. 1c) in atherosclerotic tissues with necrotic cores. Taken together, these results indicate that necroptosis indeed occurs in human atherosclerotic lesions and is correlated with necrotic core formation.

### RIPK1/RIPK3-dependent necroptosis in THP-1-derived foam cells is induced by serum starvation.

**Figure 2:**
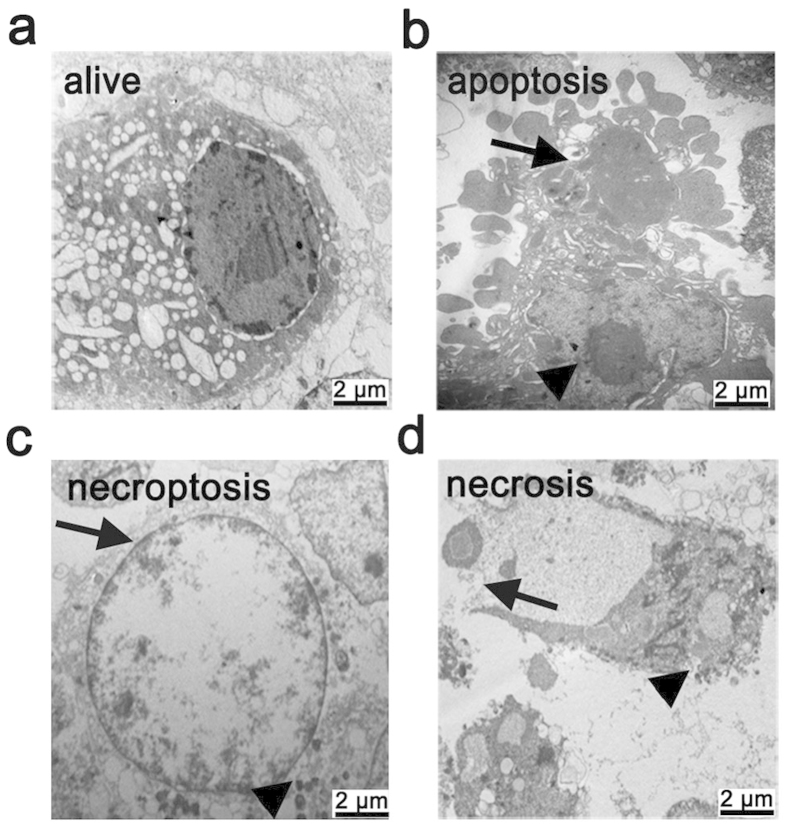
Electron microscopic characteristics of different types of cell death in foam cells. (**a**) A viable foam cell with large lipid droplets, normal cell organelles, as well as intact plasma and nuclear membranes. (**b**) Apoptotic cell showing nuclear shrinkage, chromatin condensation (triangle), and apoptotic body formation (arrow). (**c**) Necroptotic cell showing swollen nucleus (arrow), increased cell volume, disrupted cell membrane integrity, and swollen organelles (triangle). (**d**) Necrotic cell showing signs of a ruptured nucleus and plasma membrane, a loss of intracellular contents (arrow), and ruptured organelles (triangle). scale bar is 2 μm.

**Figure 3:**
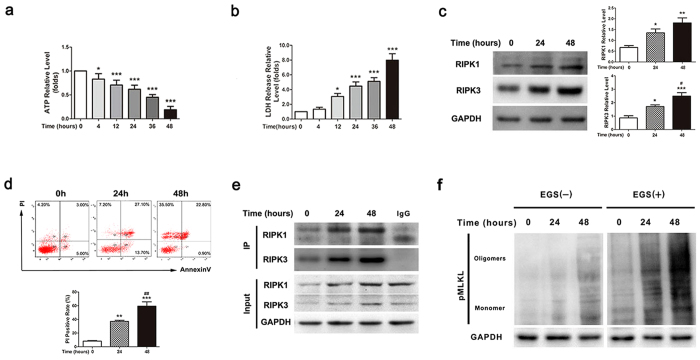
Activation of necroptosis and formation of the RIPK1-RIPK3 complex are increased after serum-free starvation in foam cells. (**a**) The reduction of intracellular ATP in THP-1 foam cells after serum deprivation. (n = 5; *P < 0.05 and ***P < 0.001 compared to the 0-h group). (**b**) The increase of LDH release from THP-1 foam cells after serum deprivation. (n = 6; *P < 0.05 and ***P < 0.001 compared to the 0-h group). (**c**) Western blotting analysis shows the elevation of RIPK1 and RIPK3 levels in foam cells undergoing serum starvation for 24 or 48 hours. In right panel, protein expression was quantified relative to GAPDH. (n = 6; *P < 0.05, **P < 0.01 and ***P < 0.001 compared to 0-h group, ^#^P < 0.05 compared to 24-h group) (**d**) Flow cytometry analysis shows the percentage of necrotic and necroptotic cells (PI-positive), after serum starvation for 24 or 48 hours. The quantitative analysis is shown in bar graphs. (n = 6; **P < 0.01 and ***P < 0.001 compared to the 0-h group; ^##^P < 0.01 compared to the 24-h group). (**e**) The increase of RIPK1-RIPK3 complex in foam cells after serum starvation, indicated by immunoprecipitation. (**f**) The lysates were incubated with or without EGS (ethylene glycol bis(succinimidyl succinate)) and subjected to SDS-PAGE separation and immunoblotting. MLKL oligomerization was detected using a MLKL phosphospecific antibody. GAPDH is shown as a loading control. The data represent means ± S.E.M.

The insufficient supply of nutrients results in foam cell death in atherosclerotic plaques^31^. To mimic the pathological process of foam cell death, we treated THP-1-derived foam cells with serum-free medium. To discern which type of cell death occurred in foam cells after serum starvation, we used TEM to observe cellular morphology. Twenty-four hours after serum deprivation, all types of cell death, including apoptosis, necroptosis, and necrosis, were observed in foam cells (Fig. 2). The morphological changes of necroptotic cells are distinct from that of apoptotic or necrotic cells. In contrast to apoptotic cells (Fig. 2b), membrane leakiness and organelle swelling were observed to occur early in necroptotic cells (Fig. 2c); but apoptotic bodies were not formed in these cells. Campared with cells undergoing uncontrolled necrosis (Fig. 2d), necroptotic cells showed swollen nuclear, increased cell volume with translucent cytoplasm (Fig. 2c); however, these cells did not display a ruptured nuclear membrane. After serum starvation, the intracellular ATP levels and the release of lactate dehydrogenase (LDH) were measured at different time points. As shown in Fig. 3a, the intracellular ATP levels significantly decreased with the extension of serum deprivation (Fig. 3a). At the same time, lactate dehydrogenase (LDH) was drastically released into culture medium (Fig. 3b). These results suggested that THP-1-derived foam cells underwent cell death in response to serum starvation. Consistent with these observations, flow cytometry analysis also showed an increased rate of cell death after serum starvation, as indicated by positive PI staining (Fig. 3d).

In addition to the increased rate of cell death, the incubation of foam cells with serum-free medium for 24 and 48 hours led to a significant elevation of RIPK1 and RIPK3 protein levels (Fig. 3c,d). More important, the level of the RIPK1-RIPK3 complex, a representative marker of necroptosis, was significantly increased over time after serum starvation (Fig. 3e). In addition, the levels of MLKL oligomerization were also significantly elevated after serum starvation (Fig. 3f). Summarily, the above results indicate that necroptosis was successfully induced in foam cells by serum deprivation.

**Figure 4:**
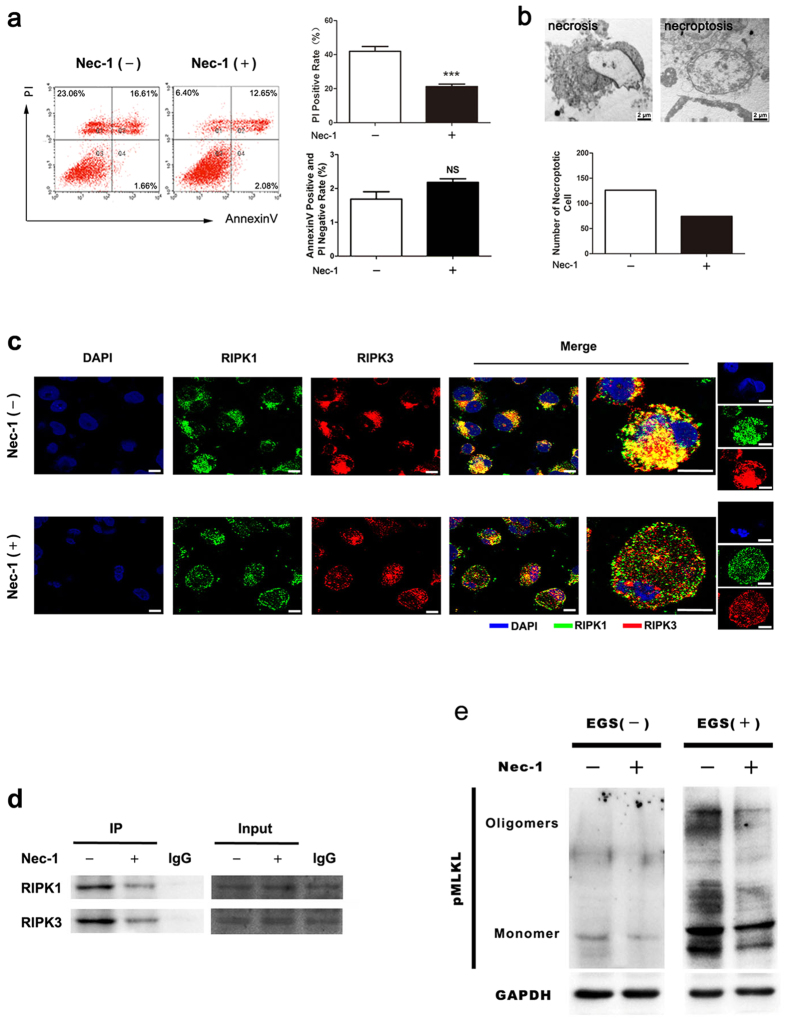
Effects of necrostatin-1 on foam cell necroptosis induced by serum starvation. (**a**) Flow cytometry analysis (left penal) shows pre-treament with Nec-1 block cell death induced by serum deprivation. The right panel shows the quantification data of flow cytometry analysis (n = 6; ***P < 0.001 compared to the control group, NS = no significant difference). (**b**) TEM photomicrograph of foam cells with or without Nec-1 treatment (upper panel). In total 200 cells counted for each sample, the numbers of necroptotic cells was recorded and is shown in the lower panel. (**c**) Immunoprecipitation assay indicates that Nec-1 treatment inhibits the formation of RIPK1-RIPK3 complex in foam cells after serum starvation. (**d**) Confocal microscopy images showing the distribution and colocalisation of RIPK1 and RIPK3 in serum-starved foam cells (scale bars = 8 μm). (**e**) The lysates were incubated with or without EGS (ethylene glycol bis(succinimidyl succinate)), subjected to SDS-PAGE separation, and analysed by immunoblotting with MLKL phosphospecific antibody. The data represent means ± S.E.M.

Since PI staining cannot distinguish necroptosis from necrosis, we next determined the percentage of necroptotic cells among the dead cells after serum starvation. To this end, we used a necroptotic-specific inhibitor, Necrostatin-1 (Nec-1), to block serum deprivation-induced necroptosis. Using Annexin V and propidium iodide (PI) staining, we determined the rates of necrosis (PI-positive) and apoptosis (PI-negative and Annexin V-positive) by flow cytometry. As shown in Fig. 4a, Nec-1 treatment dramatically reduced foam cell death from 41.94 ± 5.69% to 21.20 ± 2.90%; but had little effect on the apoptotic rate. This result suggested that around half of dead cells were attributed to necroptosis after the foam cells were deprived of serum. TEM analysis of around 200 cells also showed a similar result. As shown in Fig. 4b, Nec-1 treatment significantly decreased necroptotic cells. Furthermore, both immunofluorescence and immunoprecipitation analysis showed that Nec-1 attenuated the formation of the RIPK1-RIPK3 complex (Fig. 4c,d). In addition, Nec-1 treatment dramatically reduced the levels of MLKL oligomerization in foam cells after serum starvation (Fig. 4e).

**Figure 5:**
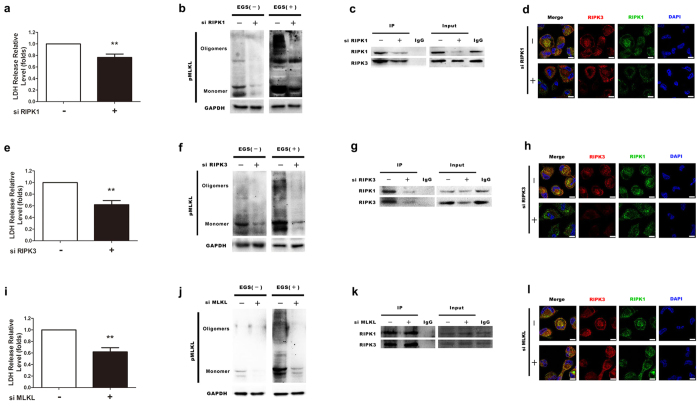
Effects of RIPK1, RIPK3 and MLKL on foam cell necroptosis induced by serum starvation. (**a**,**e**) and (i) THP-1-derived foam cells were transfected with siRNA targeting MLKL, RIPK1 and RIPK3, as indicated. The foam cell death rate was measured using an LDH assay, separately. (n = 6; *P < 0.05 and ***P < 0.001 compared to the siRNA-negative group). (**b,f**) and (**j**) MLKL phosphorylation and oligomerization was detected using an MLKL phosphospecific antibody. GAPDH was used as a loading control (bottom panel). (**c,g**) and (**k**) The lysates from cells transfected with or without siRNA were immunoprecipitated with an antibody against RIPK1, followed by western blotting analysis with an antibody against RIPK3. The input whole-cell lysates were probed with RIPK1 and RIPK3 antibodies. (**d,h**) and (**l**) Confocal microscopy images showing the distribution and colocalisation of RIPK1 and RIPK3 in serum-starved foam cells transfected or not with siRNA (scale bars = 8 μm). The data represent means ± S.E.M.

To further dissect the functional roles of RIPK1, RIPK3 and MLKL in serum deprivation-induced necroptosis, we utilised siRNAs to knockdown RIPK1, RIPK3 and MLKL, respectively. The knockdown efficiency of different siRNAs was checked with western blot analysis (Supplementary Figure f–k). According to the results of western blot, RIPK1-sh#1, RIPK3-sh#1 and MLKL-sh#2 were chosen for further experiments. As illustrated in Fig. 5a,e,b,f, knockdown of RIPK1 or RIPK3 resulted in the significant decrease of LDH release and MLKL oligomerization in serum starved foam cells. Furthermore, the formation of RIPK1-RIPK3 necrosome was blocked by knocking down either RIPK1 or RIPK3, indicated by both immunoprecipitation and immunofluorescence assays (Fig. 5c,g,d,h). These results suggest that RIPK1 and RIPK3 are required for necroptosis occurred in foam cells undergoing serum deprivation. As expected, MLKL downregulation by specific siRNA also significantly abolished LDH release and MLKL oligomerization induced by serum starvation (Fig. 5I,j); whereas did not affect the formation RIPK1-RIPK3 complex (Fig. 5kl). These data confirmed that MLKL functions as a downstream of RIPK1 and RIPK3. Summarily, it is convincing that serum starvation induces RIPK1/RIPK3-dependent necroptosis in THP-1-derived foam cells.

### ALA-mediated SDT inhibits RIPK1/RIPK3-dependent necroptosis.

**Figure 6:**
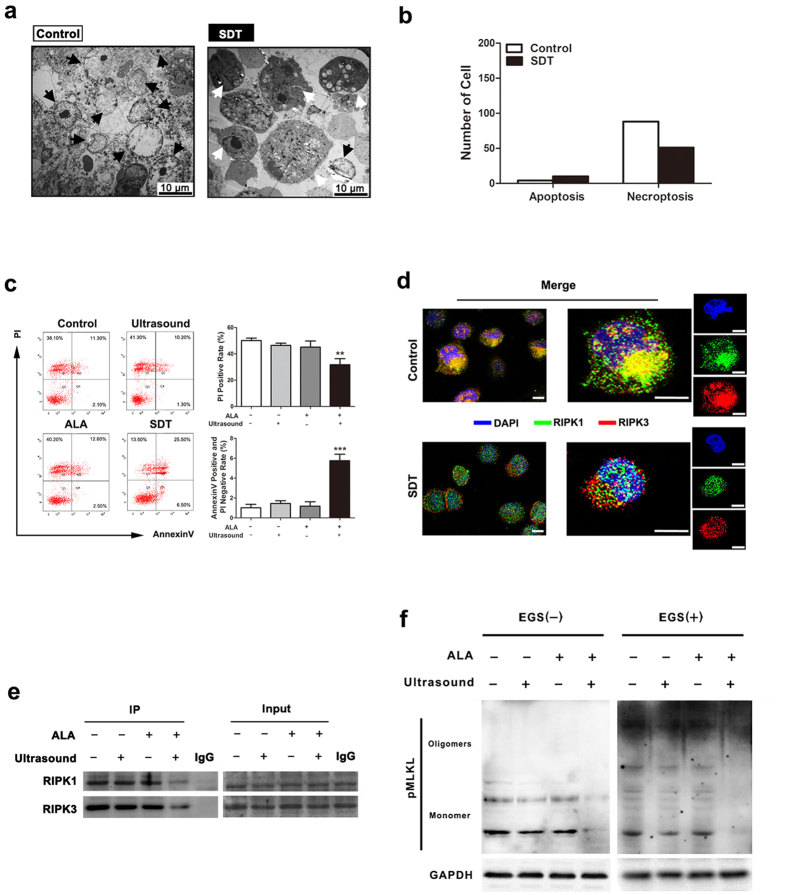
Effects of ALA-SDT on RIPK1/RIPK3-dependent necroptosis in foam cells. (**a**) ALA-SDT inhibits necroptosis in foam cells. TEM photomicrographs show the representative morphological characteristics of untreated foam cells and ALA-SDT treated foam cells (scale bars = 10 μm). The white arrow shows apoptotic cells, and necroptotic foam cells are indicated with a black arrow. (**b**) Among the 200 cells counted, the numbers of apoptotic and necroptotic cells were recorded separately for each sample. (**c**) Flow cytometry analysis (left penal) shows that ALA-SDT inhibits necroptosis, while enhaces apoptosis in foam cells. Right panel: the mean percentages of PI-positive cells and Annexin V-positive/PI-negative cells were analysed by flow cytometry. (n = 6; **P < 0.01 and ***P < 0.001 compared to the control group). (**d**) Confocal microscopy images shows that ALA-SDT inhibits the colocalisation of RIPK1 and RIPK3 in serum-starved foam cells. (scale bars = 8 μm). (**e**) ALA-SDT blocks the formation of RIPK1-RIPK3 complex in serum-starved foam cells. (**f**) ALA-SDT blocks the levels of pMLKL oligomerization in serum-starved foam cells. The data represent means ± S.E.M.

The above observations indicate the successful establishment of an *in vitro* necroptotic model, in which necroptosis is induced in foam cells by serum deprivation. Using this model, we next tested whether ALA-SDT affects necroptosis. Twelve hours after serum starvation, foam cells were treated with ALA-SDT. TEM analysis revealed that ALA-SDT significantly suppressed necroptosis induced by serum starvation; in contrast, dramatically enhanced apoptotic cells (Fig. 6ab). This result was further supported by flow cytometry analysis. The percentage of cell death (PI-positive cells) decreased from 50.08 ± 4.22% to 31.73 ± 9.24% after ALA-SDT treatment. Meanwhile, the apoptotic rate (PI-negative and Annexin V-positive) increased from 1.02 ± 0.77% to 5.75 ± 1.31% (Fig. 6c). Although ALA-SDT treatment did not alter the protein levels of RIPK1 and RIPK3 individually, it disrupted necrosome formation. ALA-SDT remarkably attenuated a number of cells with RIPK1-RIPK3 colocalisation (Fig. 6d), and levels of the RIPK1-RIPK3 complex (Fig. 6e) and MLKL oligomerization in foam cells (Fig. 6f). These results suggest that ALA-SDT switchs the cell death mechanism in foam cells from necroptosis to apoptosis by disrupting the RIPK1-RIPK3 complex.

### ALA-mediated SDT inhibits RIPK1-RIPK3 necrosome through caspase-8 activation.

**Figure 7:**
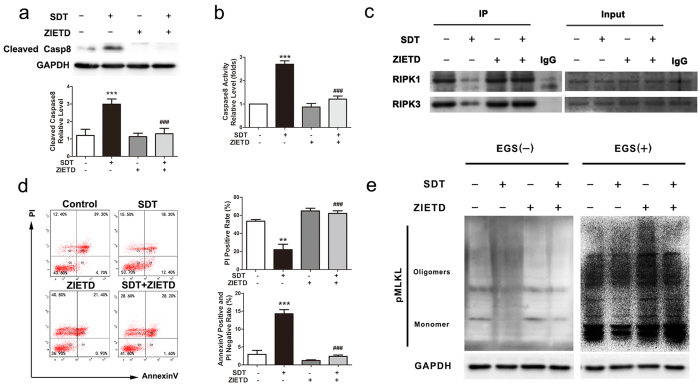
The effects of ZIETD on caspase-8 activation and RIPK1/RIPK3-dependent necroptosis in ALA-SDT-treated foam cells. (**a**) Z-IETD-fmk (ZIETD, a caspase-8 specific inhibitor, 20 μM), inhibits the increased protein levels of cleaved caspase-8 in ALA-SDT treated foam cells (upper panel). Protein expression was quantified in the lower panel relative to GAPDH. (n = 4; ***P < 0.001 compared to the control group, ^##^P < 0.01 compared to the SDT group). (**b**) ZIETD inhibits the increased protein levels of cleaved caspase-8 in ALA-SDT treated foam cells (n = 5; ***P < 0.001 compared to the control group, ^###^P < 0.001 compared to the SDT group) (**c**) ZIETD stabilizes the RIPK1-RIPK3 complex in ALA-SDT treated foam cells. (**d**) ZIETD reverses the anti-cell death effect of ALA-SDT, analysed with flow cytometry (left panel). Right panel shows the quantification data of flow cytometry analysis. (n = 6; **P < 0.01 and ***P < 0.001 compared to the control group, ^###^P < 0.001 compared to the SDT group) (**e**) ZIETD blocks the decreased levels of pMLKL oligomerization in ALA-SDT treated foam cells. The data represent means ± S.E.M.

To further explore the mechanism underlying the anti-necroptotic effect of ALA-SDT, we checked the status of caspase-8, whose activation can block the initiation of necroptosis by inhibiting the formation of RIPK1-RIPK3 complex and MLKL oligomerization. We found that ALA-SDT increased both cleaved caspase-8 levels and activity (Supplementary Figure c,d, Fig. 7a,b). To investigate whether the enhanced caspase-8 activity accounted for the anti-necroptotic effect of ALA-SDT, we pre-treated foam cells with a caspase-8-specific inhibitor, z-IETD-fmk (ZIETD), and then subjected the cells to ALA-SDT. As expected, pre-incubation with ZIETD inhibited the elevation of cleaved caspase-8 levels and caspase-8 activity in ALA-SDT treated foam cells (Fig. 7a,b). Further experiments showed that pre-treatment with ZIETD abolished the anti-necroptotic effect of ALA-SDT through stabilizing the RIPK1-RIPK3 complex (Fig. 7c,d) and reducing MLKL oligomerization (Fig. 7e). Together, our data suggest that ALA-SDT leads to the activation of caspase-8, inhibits the formation of the RIPK1-RIPK3 necrosome, decreases MLKL oligomerization, and consequently protects foam cells from necroptosis.

### Caspase-3 is an upstream regulator of caspase-8 activation in ALA-SDT-treated cells.

**Figure 8:**
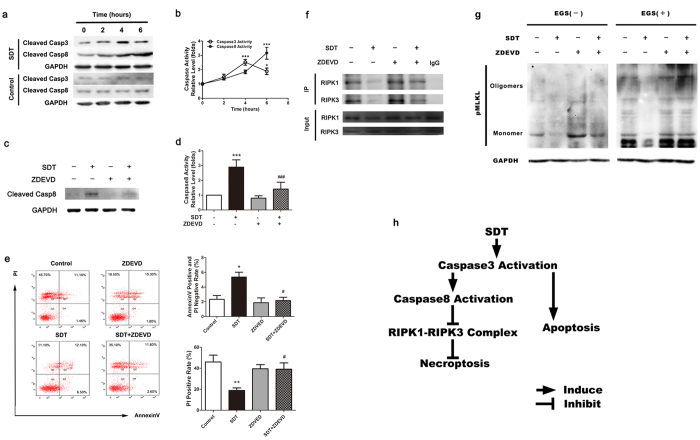
The effects of ZDEVD on caspase-8 activation and the anti-necroptotic effect of ALA-SDT in foam cells. (**a**) The changes of protein level of cleaved caspase-3/8 in foam cells at the different time point after ALA-SDT. (**b**) The dynamics of activated caspase-3/8 activity after ALA-SDT. (n = 4; *P < 0.05 and ***P < 0.001 compared to the control group). (**c**) Pretreatment with a caspase-3-specific inhibitor, z-DEVD-fmk (ZDEVD, 20 μM) inhibits the increased protein levels of cleaved caspase-8 in ALA-SDT treated foam cells. (**d**) ZDEVD blocks the increased activity of caspase-8 in ALA-SDT treated foam cells. (n = 5; ***P < 0.001 compared to the control group, ^###^P < 0.001 compared to the SDT group). (**e**) ZDEVD reverses the anti-cell death effect of ALA-SDT, analyzed with flow cytometry (left panel). The right panel shows the quantification data of flow cytometry analysis. (n = 3; *P < 0.05 and **P < 0.01 compared to the control group, ^#^P < 0.05 compared to the SDT group). (f) ZDEVD stabilizes the RIPK1-RIPK3 complex in ALA-SDT treated foam cells. (**g**) ZDEVD blocks the decreased pMLKL oligomerization levels in ALA-SDT treated foam cells. (**h**) A proposed model of the ALA-SDT induced anti-necroptotic effect. The data represent means ± S.E.M.

We next asked how caspase-8 is activated after ALA-SDT treatment. To this end, we monitored the alteration of caspase-3 and caspase-8 after ALA-SDT treatment. The levels of the active form of both caspase-3 and caspase-8 were significantly elevated at 2, 4 and 6 hours after treatment (Fig. 8a,b). However, the dynamics of their enzymatic activity were quite different. Caspase-3 activation peaked 4 hours after ALA-SDT treatment, 2 hours earlier than the maximal caspase-8 activation. This result suggested that caspase-3 may be an upstream enzyme responsible for caspase-8 activation. Indeed, pre-treatment with a caspase-3 specific inhibitor, z-DEVD-fmk (ZDEVD), abolished the caspase-8 activation in ALA-SDT-treated cells (Fig. 8c,d). Since ALA-SDT prevented foam cells from necroptosis by activating caspase-8, we explored whether ZDEVD could reverse the anti-necroptotic effect of ALA-SDT. As expected, pre-incubation with ZDEVD markedly inhibited the anti-cell death effect of ALA-SDT (Fig. 8e). Meanwhile, the enhanced apoptosis following ALA-SDT was also suppressed by this inhibitor (Fig. 8e). Immunoprecipitation assay showed that pre-incubation with ZDEVD stabilised the RIPK1-RIPK3 complex (Fig. 8f) and MLKL oligomerization (Fig. 8g). In summary, ALA-SDT exerted its anti-necroptotic effect through activating the caspase-3 and caspase-8 pathways.

## Discussion

The present studies demonstrate that necroptosis occurs in human atherosclerotic lesions and may contribute to the progression of atherosclerosis. The necroptotic markers, RIPK1, RIPK3 and the RIPK1-RIPK3 complex, are significantly increased in human atherosclerotic tissues with necrotic core. More important, we found that ALA-SDT inhibits necroptosis, while promotes apoptosis by activating caspase-3 and caspase-8 (Fig. 8h). This necroptosis to apoptosis switch suggests that ALA-SDT might be an efficient regimen for the treatment of atherosclerosis.

Necroptosis is a newly identified form of programmed cell death and displays unique morphological features that are distinct from apoptosis and necrosis^6^. Necroptosis is initiated by formation of the necrosome, of which the major component is the RIPK1-RIPK3 complex^12^,^13^. Activated RIPK3 then phosphorylates the pseudo-kinase MLKL, inducing its oligomerization and insertion into the plasma membrane and leading to necroptosis^4^,^32^. Necroptosis not only facilitates the formation of the necrotic core in atherosclerotic lesions but also drives the infiltration of inflammatory cells^12^,^33^. High rates of necroptosis in advanced atherosclerotic plaques contribute to the destabilisation of atherosclerosis and increase the risk of plaque rupture and thrombosis^8^. Nonetheless, the involvement of necroptosis in human atherosclerosis has never been experimentally evaluated or verified. In this study, to our knowledge, it is the first time to confirm necroptosis in clinical atherosclerotic lesions. First, some foam cells in human atherosclerotic plaques displayed necroptotic morphological features, including enhanced cytoplasmic translucency, swollen organelle, increased cell volume, disrupted cell membrane and intact nucleus. Second, the expression of RIPK1 and RIPK3 was higher in human atherosclerotic samples with necrotic cores compared with tissues from the same vessel without necrotic cores. Third, levels of the RIPK1-RIPK3 necrosome were increased in human atherosclerotic lesions harbouring necrotic cores. In summary, the above evidence indicates that necroptosis occurs in clinical atherosclerosis, suggesting that it may play an important role in human atherosclerotic progression.

Diverse stimuli, such as TNF-α, ox-LDL and lipopolysaccharides, in combination with caspase inhibitors, are able to trigger necroptosis in different macrophage cell lines, for instance, J774A.1, RAW264.7 and mouse peritoneal macrophages^9^,^32^,^34^. However, these stimulations cannot efficiently induce THP-1 macrophage necroptosis (data not shown). This result is in agreement with Mocarski’s observation^35^. Low energy/ATP in the centre of atherosclerotic plaques due to insufficient nutrients results into foam cell death and the formation of necrotic core^36^. According to this knowledge, we treated THP-1-derived foam cells with serum-free medium to mimic the microenvironment in atherosclerotic plaques. Serum deprivation efficiently induced necroptosis in THP-1-derived foam cells, confirmed by morphological observation and molecular characterisation. Necroptosis accounts for approximately half of cell-death events induced by serum starvation; the other half could be caused by apoptosis or unregulated necrosis mediated by unknown mechanisms. More important, Nec-1, a RIPK1 inhibitor, as well as siRNA knockdown of RIPK1, RIPK3 and MLKL, significantly blocked cell death, further confirming that necroptosis is occurred in THP-1-derived foam cells under the condition of serum deprivation. Since energy is essential for cell survival, to what extent energy deficiency drives foam cells to go to necroptosis, necrosis or apoptosis remains to be determined in the future. The induction of necroptosis by modulating ATP levels should be further optimized and would be a good model for the basic and translational studies of necroptosis.

The RIPK1-RIPK3 necrosome is a critical initiator of necroptosis and a main target for blocking necroptosis^32^,^37^. Activated caspase-8 prevents necroptosis by blocking formation of the RIPK1-RIPK3 complex and, subsequently, MLKL oligomerization^38^. In the present study, caspase-8 activation, disruption of the RIPK1-RIPK3 complex, and reduced MLKL oligomerization were observed in ALA-SDT-treated cells. In addition, a caspase-8-specific inhibitor (ZIETD) reversed the anti-necroptotic effect of ALA-SDT, suggesting that the activation of caspase-8 plays a critical role in the anti-necroptotic effects of ALA-SDT. More important, ALA-SDT enhanced apoptosis while inhibiting necroptosis. In contrast to necroptosis and necrosis, apoptotic cells release anti-inflammatory factors, exhibit reduced inflammation reactions and result in little damage to surrounding tissues. Preventing necroptosis by initiating apoptosis may explain, at least in part, the benefit of ALA-SDT in atherosclerosis treatment.

Apoptosis and necroptosis share several common molecular pathways, implying that these two types of cell death may be two sides of the same coin and could be switched from one to the other under certain conditions^14^. Necroptosis occurs when apoptotic pathway is impaired. For instance, caspase-3 specific and pan-caspase inhibitors induce cell necroptosis^39^,^40^. The relationship between apoptosis and necroptosis is still fragmentary and elusive. It has been proposed that apoptosis has an antagonistic effect on necroptosis. This hypothesis is supported by a genetic study, in which RIPK3 deficiency rescues embryonic lethality induced by caspase-8 or FADD knockout^41^,^42^. Our studies also corroborate this hypothesis. ALA-SDT activated the caspase-3 and caspase-8 pathways in foam cells, initiated apoptosis, and thereby inhibited necroptosis. Additionally, caspase-3 and caspase-8 inhibitors reversed the anti-necroptotic effect of ALA-SDT, suggesting that caspase-3 and caspase-8 may mediate the switch between apoptosis and necroptosis.

Verification of necroptosis in human atherosclerotic plaques suggests its potential role in the pathogenesis and development of atherosclerosis. The inhibition of necroptosis mitigates pathological changes in multiple disease models^34^,^43^. For instance, the RIPK1 inhibitor, Nec-1 or RIPK3-deficiency tremendously benefits inflammatory diseases *in vivo*, such as myocardial infarction and atherosclerosis^34^,^43^. The RIPK3 inhibitors GSK’843 and GSK’872 are effective in alleviating liver injury^44^. ALA-SDT, in our study, is another methodology to inhibit necroptosis while to enhance apoptosis, implying its translational potential for the treatment of necroptosis-related diseases, including atherosclerosis. Next, it would be necessary to confirm the anti-necroptotic effect of ALA-SDT in atherosclerotic animal model.

## Methods

### Reagents

5-Aminolevulinic acid (ALA) and phorbol 12-myristate 13-acetate (PMA) were purchased from Sigma-Aldrich (St Louis, MO, USA). Necrostatin-1 (Nec-1), z-DEVD-fmk (ZDEVD) and z-IETD-fmk (ZIETD) were from Santa Cruz Biotechnology (CA). Cu-oxidised LDL (ox-LDL) and red fluorescent marked DiI-ox-LDL were both purchased from Peking Union-Biology Co. Ltd (Beijing, China). Hoechst-33342, DAPI, the ATP assay kit, the lactate dehydrogenase (LDH) release assay, and the caspase-3 and caspase-8 activity assay kit were obtained from Beyotime Institute of Biotechnology (Jiangsu, China). The ApoAlert Annexin V-FITC kit was purchased from BD Biosciences (Franklin Lakes, NJ, USA). Other drugs and chemicals used in this study were obtained from Sigma-Aldrich. EGS (ethylene glycol bis(succinimidyl succinate)) was obtained from Thermo Scientific.

### Cell cultures

THP-1 monocytes are a human monocyte line (American Type Culture Collection, Manassas, VA, USA). The cells were cultured in RPMI 1640 medium (HyClone, Logan, UT, USA) containing 10% foetal bovine serum (FBS) (HyClone, Logan, UT, USA), 20 μg/mL penicillin and 20 μg/mL streptomycin (Sigma-Aldrich, St Louis, MO, USA), and maintained at 37 °C in a humidified atmosphere containing 5% CO_2_. To differentiate THP-1 monocytes into macrophages, they were seeded onto cover slips in 35-mm Petri dishes at a density of 0.5 × 10^6^ cells/mL and treated with 100 ng/mL PMA for 72 hours. The macrophages were incubated with 100 μg/mL ox-LDL for 48 hours in serum-free RPMI 1640 to form foam cells. The inhibitors ZIETD, ZDEVD and Nec-1 were used at appropriate concentrations (ZDEVD, 20 μM; ZIETD, 20 μM; Nec-1, 20 μM).

### RNA interference

Two different siRNAs against RIPK1, RIPK3 and MLKL,respectively, and a negative control siRNA were designed by GenePharma Co.,Ltd. (Shanghai, China), according to a previously described protocol. ThesiRNA sequences were as follows: RIPK1-sh#1^45^: 5′-GCACAAATACGAACTTCAA-3′, RIPK1-sh#2^46^: 5′-CCACUAGUCUGACGGAUAA-3′, RIPK3-sh#1^12^: 5′-UAACUUGACGCACGACAUCAGGCUGUU-3′, RIPK3-sh#2^12^: 5′-GCAGUUGUAUAUGUUAACGAGCGGUCG-3′. MLKL-sh#1^47^: 5′-GCGTATATTTGGGATTTGCAT-3′, and MLKL-sh#2^46^: 5′-CAAACUUCCUGGUAACUCA-3′. The siRNAknockdowns were performed using X-tremeGENE siRNA Transfection Reagent (Roche) according to the manufacturer’s protocol. Briefly, 1 × 10^6^ cells wereseeded in 35-mm Petri dishes to obtain approximately 80–90% confluence. Two hundred microlitres of a mixture containing siRNA (2 μg for each) and transfection reagents (12 μL for each) was added to the cells for 6 h at 37 °C, and then replaced with fresh culture medium. The levels of RIPK1, RIPK3 and MLKL were determined using western blotting analysis.

### Sonodynamic therapy treatment

The SDT equipment used in this study, including an ultrasonic generator, transducer and power amplifier, was assembled by the Harbin Institute of Technology (Harbin, China). The homemade ultrasonic transducer (diameter: 3.5 cm; resonance frequency: 1.0 MHz; duty factor: 10%; repetition frequency: 100 Hz) was placed in a water bath 30 cm below the cells. The ultrasonic intensity used was 0.4 W/cm^2^, as measured by a hydrophone (Onda Corp, Sunnyvale, CA, USA). THP-1-derived foam cells (0.5 × 10^6^ cells/mL) were seeded in 35-mm Petri dishes, consistent with the size of the therapeutic instrument probe, before SDT treatment. The cells were incubated with 1 mM ALA (Sigma-Aldrich, St Louis, MO, USA) in the dark for 6 hours prior to treatment with the SDT equipment.

### Human sample collection

We performed an autopsy of 12 individuals aged 36 to 58 years from the Department of Forensic Medicine of Harbin Medical University. In addition, 6 human tissue samples were collected from atherosclerotic patients of 40–60 years of age by carotid endarterectomy (CEA) at the First Affiliated Hospital of Harbin Medical University. Informed written consent was obtained from all patients. The study protocol conformed to the Ethical Guidelines of the 1975 Declaration of Helsinki, and was approved by the First Affiliated Hospital of Harbin Medical University (People’s Republic of China) Human Ethics Committee.

### HE staining

All tissues were sliced at 5 μm, mounted on glass slides coated with poly L-lysine, and subjected to haematoxylin and eosin staining according to routine histopathological methods. Histopathological changes were observed under a light microscope.

### Transmission electron microscopy

The cells were rinsed three times with PBS and then fixed with 2.5% glutaraldehyde in 0.1 M phosphate buffer saline (PBS, pH 7.3) for 1 hour at room temperature. Next, the prepared samples were determined by transmission electron microscopy (TEM) (JEM-1220, Japan) according to conventional methods. For quantitative analysis of necrotic foam cells. Total 200 cells were counted, and the number of necrotic foam cells was recorded.

### Western blotting analysis

Proteins were extracted with RIPA buffer from foam cells 4 hours after SDT treatment. The proteins were separated on 7.5%, 10%, 12% or 15% SDS-PAGE gels and transferred to a nitrocellulose membrane. The membranes were incubated with primary antibodies, including those against cleaved caspase-3, cleaved caspase-8, RIPK1, RIPK3, MLKL, pMLKL and GAPDH (Cell Signaling Technology, Inc., USA). Antibody labelling was detected using a ChemiDoc MP imaging system (Universal Hood III, Bio-Rad Laboratories, Inc., USA) with BeyoECL Plus (Beyotime, Jiangsu, China), according to the manufacturer’s instructions. The labelling was analysed with Image Lab software (version 4.1, Bio-Rad).

### MTT assay

Cell viability was determined with MTT assay (Sigma-Aldrich, St Louis, MO, USA). Cells (1 × 10^5^ cells/mL) were seeded in 96-well culture plates. Ox-LDL was added to the treatment group for 4 hours at concentration of 50–200 μg/mL. The control group was incubated with RPMI 1640 medium without ox-LDL. Cytotoxicity was determined by adding 10 μL MTT solution (5 mg/ml in PBS) to each well, and the mixture was incubated for 4 hours at 37 °C in a CO_2_ incubator. The formed formazan crystals were dissolved in a solution of 100 μl 10% sodium dodecyl sulphate (SDS) and 0.01 M HCl. The absorbance was measured at 570 nm using a Model 680 Microplate Reader. The macrophage viability of the treated samples was then analysed in comparison to the control.

### ATP assay

For the ATP assay, a luminescence-based commercial kit was obtained from Beyotime Institute of Biotechnology (Jiangsu, China). Intracellular ATP levels were measured at suitable time points in serum-free RPMI 1640 according to the manufacturer. Luminescence was measured using a Glomax 20/20 luminometer (Promega). The ATP level was determined as a multiple of that observed in the untreated control group.

### LDH release assay

Foam cell necrosis was assessed using LDH assay kit. Cells were seeded in 35-mm Petri dishes and measured at suitable time points in serum-free RPMI 1640 according to the manufacturer. The absorbance was recorded at 490 nm using a micro-plate reader. The basal level of LDH release was determined with 50 μL supernatant from cells cultured with medium. The calculation of LDH release was as follows: (LDH release from treated group minus basal release) divided by (maximal LDH release minus basal release).

### Flow cytometry analysis

We stained cells using Annexin V-FITC and PI Detection Kit (BD Pharmingen, USA) prior to the analysis with flow cytometry. Briefly, cells were seeded in 35-mm Petri dishes at a density of 1 × 10^6^ cells per well. The cells were cultured in 2 mL phenol red-free medium containing 10% FBS, and necroptosis was then induced as described above. Then, the foam cells were measured at suitable time points according to the manufacturer. The results were analysed using BD FACSDiva Software v7.0 (Becton-Dickinson, USA). For proper statistical analysis, more than 10000 cells per group were counted, and each assessment was repeated three times.

### Immunofluorescence

The cells were fixed with 4% paraformaldehyde for 10 min at room temperature and then permeabilized with 0.2% Triton X-100 for 10 min at room temperature. The cells were blocked with 3% BSA (Sigma-Aldrich, St Louis, MO, USA) at room temperature for 30 minutes. Next, the cells were incubated with anti-RIPK1 (Cell Signaling Technology, Inc., USA) and anti-RIPK3 (Santa Cruz Biotechnology, CA) antibodies for 2 hours at 37 °C. Then, the cells were rinsed with PBS and incubated with Dylight 594 and DyLight 488 AffiniPure secondary antibodies (EarthOx, San Francisco, CA, USA) for 1 hour. Next, the cells were counterstained with DAPI for 15 minutes at 37 °C in the dark. After being washed with PBS, the cells were visualised by confocal microscopy (Carl Zeiss LSM700).

### Caspase activity analysis

The cells were collected by trypsinisation, rinsed twice with PBS, re-suspended in lysis buffer supplied by the caspase-3 and/or caspase-8 activity assay kit and incubated on ice for 15 minutes. After centrifugation, the supernatant was measured for protein concentration using the Bradford method. Total protein (0.1 mg) was used for the caspase activity assay, with Ac-DEVD-pNA or Ac-IETD-pNA as the substrates of caspase-3 and caspase-8, respectively. Absorbance at 405 nm resulting from the production of pNA was continuously recorded using a Model 680 Microplate Reader after incubation for 2 hours at 37 °C.

### Statistical analysis

All the experiments were repeated at least 3 times independently. The data represent means ± S.E.M. SAS 9.1 (SAS Institute Inc., Cary, NC) were used for statistical analysis. The differences between groups were determined by one-way analysis of variance (ANOVA) followed by the Bonferroni post hoc test, when appropriate. For analysis of statistical difference between two groups, a Student’s two-tailed t-test was applied. Differences among groups were determined by variance (ANOVA), and a Bonferroni post hoc test. P-values of 0.05 or less were considered statistically significant.

## Additional Information

**How to cite this article**: Tian, F. *et al.* 5-Aminolevulinic Acid-Mediated Sonodynamic Therapy Inhibits RIPK1/RIPK3-Dependent Necroptosis in THP-1-Derived Foam Cells. *Sci. Rep.* **6**, 21992; doi: 10.1038/srep21992 (2016).

## Supplementary Material

Supplementary Information

## References

[b1] Tabas I. Macrophage death and defective inflammation resolution in atherosclerosis. Nat Rev Immunol 10, 36–46 (2010). 19960040 10.1038/nri2675 PMC2854623

[b2] Li J. *et al.* Interferon-alpha priming promotes lipid uptake and macrophage-derived foam cell formation: a novel link between interferon-alpha and atherosclerosis in lupus. Arthritis Rheum 63, 492–502 (2011). 21280004 10.1002/art.30165

[b3] Walsh C. M. Grand challenges in cell death and survival: apoptosis vs. necroptosis. Front Cell Dev Biol 2, 3 (2014). 25364712 10.3389/fcell.2014.00003 PMC4206982

[b4] O’Donnell M. A. *et al.* Caspase 8 inhibits programmed necrosis by processing CYLD. Nat Cell Biol 13, 1437–1442 (2011). 22037414 10.1038/ncb2362 PMC3229661

[b5] Zender L. *et al.* Caspase 8 small interfering RNA prevents acute liver failure in mice. Proc Natl Acad Sci USA 100, 7797–7802 (2003). 12810955 10.1073/pnas.1330920100 PMC164667

[b6] Vandenabeele P., Galluzzi L., Vanden Berghe T. & Kroemer G. Molecular mechanisms of necroptosis: an ordered cellular explosion. Nat Rev Mol Cell Biol 11, 700–714 (2010). 20823910 10.1038/nrm2970

[b7] Kroemer G. *et al.* Classification of cell death: recommendations of the Nomenclature Committee on Cell Death. Cell Death Differ 12 Suppl 2, 1463–1467 (2005). 16247491 10.1038/sj.cdd.4401724

[b8] Lin J. *et al.* A role of RIP3-mediated macrophage necrosis in atherosclerosis development. Cell Rep 3, 200–210 (2013). 23333278 10.1016/j.celrep.2012.12.012

[b9] Zhou W. & Yuan J. SnapShot: Necroptosis. Cell 158, 464–464 e461 (2014). 25036639 10.1016/j.cell.2014.06.041

[b10] Gao X. *et al.* PEDF and PEDF-derived peptide 44mer protect cardiomyocytes against hypoxia-induced apoptosis and necroptosis via anti-oxidative effect. Sci Rep 4, 5637 (2014). 25012184 10.1038/srep05637 PMC4092347

[b11] Wu L. *et al.* 1,2-benzisothiazol-3-one derivatives as a novel class of small-molecule caspase-3 inhibitors. Bioorg Med Chem 22, 2416–2426 (2014). 24656804 10.1016/j.bmc.2014.03.002

[b12] Cho Y. S. *et al.* Phosphorylation-driven assembly of the RIP1-RIP3 complex regulates programmed necrosis and virus-induced inflammation. Cell 137, 1112–1123 (2009). 19524513 10.1016/j.cell.2009.05.037 PMC2727676

[b13] Trichonas G. *et al.* Receptor interacting protein kinases mediate retinal detachment-induced photoreceptor necrosis and compensate for inhibition of apoptosis. Proc Natl Acad Sci USA 107, 21695–21700 (2010). 21098270 10.1073/pnas.1009179107 PMC3003048

[b14] Han J., Zhong C. Q. & Zhang D. W. Programmed necrosis: backup to and competitor with apoptosis in the immune system. Nat Immunol 12, 1143–1149 (2011). 22089220 10.1038/ni.2159

[b15] Kaiser W. J. *et al.* RIP1 suppresses innate immune necrotic as well as apoptotic cell death during mammalian parturition. Proc Natl Acad Sci USA 111, 7753–7758 (2014). 24821786 10.1073/pnas.1401857111 PMC4040608

[b16] You Z. *et al.* Necrostatin-1 reduces histopathology and improves functional outcome after controlled cortical impact in mice. J Cereb Blood Flow Metab 28, 1564–1573 (2008). 18493258 10.1038/jcbfm.2008.44 PMC2831087

[b17] Bonapace L. *et al.* Induction of autophagy-dependent necroptosis is required for childhood acute lymphoblastic leukemia cells to overcome glucocorticoid resistance. J Clin Invest 120, 1310–1323 (2010). 20200450 10.1172/JCI39987 PMC2846044

[b18] Feng S. *et al.* Cleavage of RIP3 inactivates its caspase-independent apoptosis pathway by removal of kinase domain. Cell Signal 19, 2056–2067 (2007). 17644308 10.1016/j.cellsig.2007.05.016

[b19] Northington F. J. *et al.* Necrostatin decreases oxidative damage, inflammation, and injury after neonatal HI. J Cereb Blood Flow Metab 31, 178–189 (2011). 20571523 10.1038/jcbfm.2010.72 PMC3049482

[b20] Degterev A. *et al.* Chemical inhibitor of nonapoptotic cell death with therapeutic potential for ischemic brain injury. Nat Chem Biol 1, 112–119 (2005). 16408008 10.1038/nchembio711

[b21] Yumita N., Nishigaki R., Umemura K. & Umemura S. Hematoporphyrin as a sensitizer of cell-damaging effect of ultrasound. Jpn J Cancer Res 80, 219–222 (1989). 2470713 10.1111/j.1349-7006.1989.tb02295.x PMC5917717

[b22] Trendowski M. The promise of sonodynamic therapy. Cancer Metastasis Rev 33, 143–160 (2014). 24346159 10.1007/s10555-013-9461-5

[b23] Costley D. *et al.* Treating cancer with sonodynamic therapy: a review. Int J Hyperthermia 31, 107–117 (2015). 25582025 10.3109/02656736.2014.992484

[b24] Wood A. K. & Sehgal C. M. A review of low-intensity ultrasound for cancer therapy. Ultrasound Med Biol 41, 905–928 (2015). 25728459 10.1016/j.ultrasmedbio.2014.11.019 PMC4362523

[b25] Wang X. *et al.* Sonodynamic and photodynamic therapy in advanced breast carcinoma: a report of 3 cases. Integr Cancer Ther 8, 283–287 (2009). 19815599 10.1177/1534735409343693

[b26] Inui T. *et al.* Case report: A breast cancer patient treated with GcMAF, sonodynamic therapy and hormone therapy. Anticancer Res 34, 4589–4593 (2014). 25075104

[b27] Li Z. *et al.* Rapid stabilisation of atherosclerotic plaque with 5-aminolevulinic acid-mediated sonodynamic therapy. Thromb Haemost 114, 793–803 (2015). 26179778 10.1160/TH14-12-1030

[b28] Wang H. *et al.* The predominant pathway of apoptosis in THP-1 macrophage-derived foam cells induced by 5-aminolevulinic acid-mediated sonodynamic therapy is the mitochondria-caspase pathway despite the participation of endoplasmic reticulum stress. Cell Physiol Biochem 33, 1789–1801 (2014). 24923653 10.1159/000362958

[b29] Guo S. *et al.* Apoptosis of THP-1 macrophages induced by protoporphyrin IX-mediated sonodynamic therapy. Int J Nanomedicine 8, 2239–2246 (2013). 23818780 10.2147/IJN.S43717 PMC3693824

[b30] Festjens N., Vanden Berghe T., Cornelis S. & Vandenabeele P. RIP1, a kinase on the crossroads of a cell’s decision to live or die. Cell Death Differ 14, 400–410 (2007). 17301840 10.1038/sj.cdd.4402085

[b31] Leppanen O. *et al.* ATP depletion in macrophages in the core of advanced rabbit atherosclerotic plaques *in vivo*. Atherosclerosis 188, 323–330 (2006). 16405894 10.1016/j.atherosclerosis.2005.11.017

[b32] Jouan-Lanhouet S. *et al.* Necroptosis, *in vivo* detection in experimental disease models. Semin Cell Dev Biol 35, 2–13 (2014). 25160988 10.1016/j.semcdb.2014.08.010

[b33] Newton K. RIPK1. and RIPK3: critical regulators of inflammation and cell death. Trends Cell Biol 25, 347–353 (2015). 25662614 10.1016/j.tcb.2015.01.001

[b34] Sawai H. Characterization of TNF-induced caspase-independent necroptosis. Leuk Res 38, 706–713 (2014). 24773756 10.1016/j.leukres.2014.02.002

[b35] Omoto S. *et al.* Suppression of RIP3-dependent necroptosis by human cytomegalovirus. J Biol Chem 290, 11635–11648 (2015). 25778401 10.1074/jbc.M115.646042 PMC4416866

[b36] Dominic E. A. *et al.* Mitochondrial cytopathies and cardiovascular disease. Heart 100, 611–618 (2014). 24449718 10.1136/heartjnl-2013-304657

[b37] Li J. *et al.* The RIP1/RIP3 necrosome forms a functional amyloid signaling complex required for programmed necrosis. Cell 150, 339–350 (2012). 22817896 10.1016/j.cell.2012.06.019 PMC3664196

[b38] Ofengeim D. *et al.* Activation of necroptosis in multiple sclerosis. Cell Rep 10, 1836–1849 (2015). 25801023 10.1016/j.celrep.2015.02.051 PMC4494996

[b39] Sawai H. Differential effects of caspase inhibitors on TNF-induced necroptosis. Biochem Biophys Res Commun 432, 451–455 (2013). 23410748 10.1016/j.bbrc.2013.01.126

[b40] Galluzzi L. & Kroemer G. Necroptosis: a specialized pathway of programmed necrosis. Cell 135, 1161–1163 (2008). 19109884 10.1016/j.cell.2008.12.004

[b41] Kaiser W. J. *et al.* RIP3 mediates the embryonic lethality of caspase-8-deficient mice. Nature 471, 368–372 (2011). 21368762 10.1038/nature09857 PMC3060292

[b42] Kuang A. A., Diehl G. E., Zhang J. & Winoto A. FADD is required for DR4- and DR5-mediated apoptosis: lack of trail-induced apoptosis in FADD-deficient mouse embryonic fibroblasts. J Biol Chem 275, 25065–25068 (2000). 10862756 10.1074/jbc.C000284200

[b43] Dannappel M. *et al.* RIPK1 maintains epithelial homeostasis by inhibiting apoptosis and necroptosis. Nature 513, 90–94 (2014). 25132550 10.1038/nature13608 PMC4206266

[b44] Ribas J. *et al.* 7-Bromoindirubin-3’-oxime induces caspase-independent cell death. Oncogene 25, 6304–6318 (2006). 16702956 10.1038/sj.onc.1209648

[b45] Zhu G. *et al.* Expression of the RIP-1 gene and its role in growth and invasion of human gallbladder carcinoma. Cell Physiol Biochem 34, 1152–1165 (2014). 25277242 10.1159/000366328

[b46] Sun L. *et al.* Mixed lineage kinase domain-like protein mediates necrosis signaling downstream of RIP3 kinase. Cell 148, 213–227 (2012). 22265413 10.1016/j.cell.2011.11.031

[b47] Zhao J. *et al.* Mixed lineage kinase domain-like is a key receptor interacting protein 3 downstream component of TNF-induced necrosis. Proc Natl Acad Sci USA 109, 5322–5327 (2012). 22421439 10.1073/pnas.1200012109 PMC3325682

