# Supplementary material for: 5-Aminolevulinic Acid-Mediated Sonodynamic Therapy Inhibits RIPK1/RIPK3-Dependent Necroptosis in THP-1-Derived Foam Cells
Source: Sci Rep. 2016 Feb 25;6:21992. doi: 10.1038/srep21992 (PMC4766406; doi:10.1038/srep21992)
Supplement: Supplementary Information [file srep21992-s1.doc]

**5-aminolevulinic Acid Mediated Sonodynamic Therapy Inhibits RIPK1/RIPK3-Dependent Necroptosis in THP-1-Derived Foam Cells**

Fang Tian^1^, Jianting Yao^1^, Meng Yan^2^, Xin Sun^1^, Wei Wang^1^, Weiwei Gao^1^, Zhen Tian^2^, Shuyuan Guo^1^, Zengxiang Dong^1^, Bicheng Li^1^, Tielei Gao^5^, Peng Shan^6^, Bing Liu^6^, Haiyang Wang^6^, Jiali Cheng^1^, Qianping Gao^1^, Zhiguo Zhang^4^, Wenwu Cao^4,7^, Ye Tian^1-3^*

**Supplementary Figures**


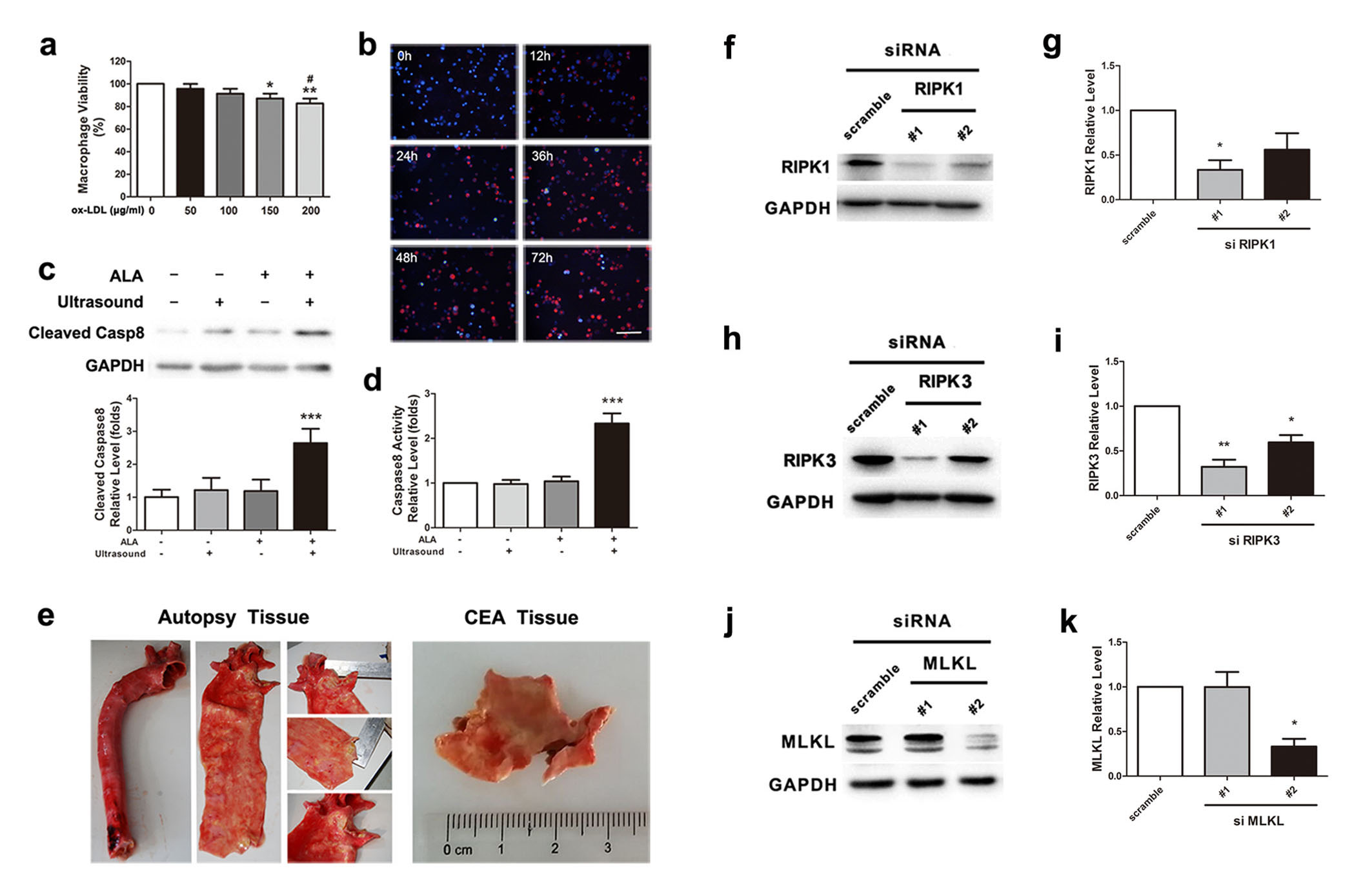


**Figure Legends**

**Supplementary Figure a:** THP-1 macrophages were exposed to ox-LDL at the indicated concentrations (0-200 μM), and cell viability was measured by MTT assays. (Abbreviation: *P < 0.05 and **P < 0.01 compared to the 0 h group). The values represent the means ± SEM from three independent experiments (n ≥ 3).
**Supplementary Figure b:** Fluorescent photomicrograph of macrophages after incubation for the indicated times with red fluorescent marked DiI-ox-LDL (100 μg/ml) in serum-free RPMI 1640 medium. Cell nuclei were stained with Hoechst-33342.
**Supplementary Figure c:** The cells were divided into untreated control, ultrasound alone, ALA alone, and SDT groups. Total cell lysates were immunoblotted and probed with antibodies against cleaved caspase-8 protein. The cells were treated with ALA-SDT and lysed after 6 h. Protein expression was quantified with respect to GAPDH. (Abbreviation: **P < 0.01 compared to the control group). The values represent the means ± SEM from three independent experiments (n ≥ 3).
**Supplementary Figure d:** The activation of caspase-8 was determined using an activated caspase-8 assay after ALA-SDT treatment for 6 h. (Abbreviation: **P < 0.01 compared to the control group). The values represent the means ± SEM from three independent experiments (n≥ 3).
**Supplementary Figure e:** Autopsy and CEA atherosclerotic plaque samples chosen in this study.

**Supplementary Figure f, h and j:** Representative western blot of RIPK1, RIPK3 and MLKL following siRNA treatment, respectively.

**Supplementary Figure g, i and k:** Quantitative measurements of level of RIPK1, RIPK3 and MLKL silencing as detected by Western blot, respectively.
